# Establishment and validation of nomogram models for overall survival and cancer-specific survival in spindle cell sarcoma patients

**DOI:** 10.1038/s41598-023-50401-z

**Published:** 2023-12-27

**Authors:** Weihui Qi, Yanyun Ren, Huang Wang, Yue Wan, Dong Wang, Jun Yao, Hao Pan

**Affiliations:** 1 Department of Orthopaedics, Hangzhou TCM Hospital Affiliated to Zhejiang Chinese Medical University, Hangzhou, 310000 Zhejiang People’s Republic of China; 2 Department of Orthopaedics, Hangzhou Ding Qiao Hospital, Hangzhou, China; 3 Department of Stomatology, No. 903 Hospital of PLA, Hangzhou, China

**Keywords:** Cancer epidemiology, Cancer therapy, Sarcoma

## Abstract

Spindle cell sarcoma (SCS) is rare in clinical practice. The objective of this study was to establish nomograms to predict the OS and CSS prognosis of patients with SCS based on the Surveillance, Epidemiology, and End Results (SEER) database. The data of patients with SCS between 2004 and 2020 were extracted from the SEER database and randomly allocated to a training cohort and a validation cohort. Univariate and multivariate Cox regression analyses were used to screen for independent risk factors for both overall survival (OS) and cancer-specific survival (CSS). Nomograms for OS and CSS were established for patients with SCS based on the results of multivariate Cox analysis. Then, we validated the nomograms by the concordance index (C-index), receiver operating characteristic (ROC) curves, calibration curves, and decision curve analysis (DCA). Finally, Kaplan‒Meier curves and log-rank tests were applied to compare patients with SCS at three different levels and in different treatment groups. A total of 1369 patients with SCS were included and randomly allocated to a training cohort (n = 1008, 70%) and a validation cohort (n = 430, 30%). Age, stage, grade, tumour location, surgery, radiation and diagnosis year were found to be independent prognostic factors for OS by Cox regression analysis, while age, stage, grade, tumour location and surgery were found to be independent prognostic factors for CSS. The nomogram models were established based on the results of multivariate Cox analysis for both OS and CSS. The C-indices of the OS model were 0.76 and 0.77 in the training and validation groups, respectively, while they were 0.76 and 0.78 for CSS, respectively. For OS, the 3- and 5-year AUCs were 0.801 and 0.798, respectively, in the training cohort and 0.827 and 0.799, respectively, in the validation cohort; for CSS, they were 0.809 and 0.786, respectively, in the training cohort and 0.831 and 0.801, respectively, in the validation cohort. Calibration curves revealed high consistency in both OS and CSS between the observed survival and the predicted survival. In addition, DCA was used to analyse the clinical practicality of the OS and CSS nomogram models and revealed that they had good net benefits. Surgery remains the main treatment method for SCS patients. The two nomograms we established are expected to accurately predict the personalized prognosis of SCS patients and may be useful for clinical decision-making.

## Introduction

Sarcoma is a rare heterogeneous tumour that mainly occurs in the embryonic mesoderm^1^, and the estimated overall incidence is 6.2/100,000/year^2^. There are more than 50 different histological subtypes of soft tissue sarcomas alone, and they have different clinical and biological characteristics^3^. Spindle cell sarcoma is an extremely rare subtype of sarcoma^4,5^. The most common primary site is the subcutaneous soft tissue of the limbs and trunk, but cases involving various organs of the body, such as the heart^6^, seminal vesicles^7^, pancreas^8^, gallbladder^9^, adrenal gland^10^, and tongue^11^, have been reported.

Spindle cell lesions encompass a heterogeneous group of tumours that range from benign to borderline and malignant tumours. Benign lesions are more common than malignant tumours are, and immunohistochemistry and molecular testing aid accurate diagnosis. Surgery and radiotherapy/chemotherapy based on different pathological types and clinical stages constitute a worldwide consensus in the treatment of sarcoma^12–15^. Due to the extremely low proportion of cases of spindle cell sarcoma among sarcomas, there have been no large-scale reports of its diagnosis or treatment; moreover, only a few case reports exist, and there are considerable differences in clinical manifestations due to differences in the types of primary tumour sites.

The National Cancer Institute’s Surveillance, Epidemiology, and End Results (SEER) database, which is the largest cancer registry and has multiple quality control measures, offers a unique opportunity to perform detailed analyses of the incidence and survival of patients with rare tumours^16,17^. In recent years, nomograms have been widely used as prediction methods in oncology^18–20^. Lei Feng et al. reported independent risk factors for OS and CSS in patients with SCS through the Seer database^21^. There is no application of nomograms for predicting the prognosis of SCS patients. In the present research, using a large dataset from the Surveillance, Epidemiology and End Results (SEER) database, we aimed to establish a nomogram to predict the OS and CSS prognosis of patients with SCS. This scoring system can help clinical doctors make more appropriate clinical decisions; and we also evaluated the impact of combined treatment on the prognosis of SCS patients.

## Materials and methods

### Data and patients

The data were obtained from the Surveillance, Epidemiology and End Results database. The SEER database includes sociodemographic characteristics, clinical factors, tumour staging, pathological variables, surgical and chemoradiotherapy methods, and prognostic information; this information is publicly accessible, and the author obtained permission. SEER* Stat software (version 8.4.1- March 29, 2023, SEER* Stat software) was used.

**Figure 1:**
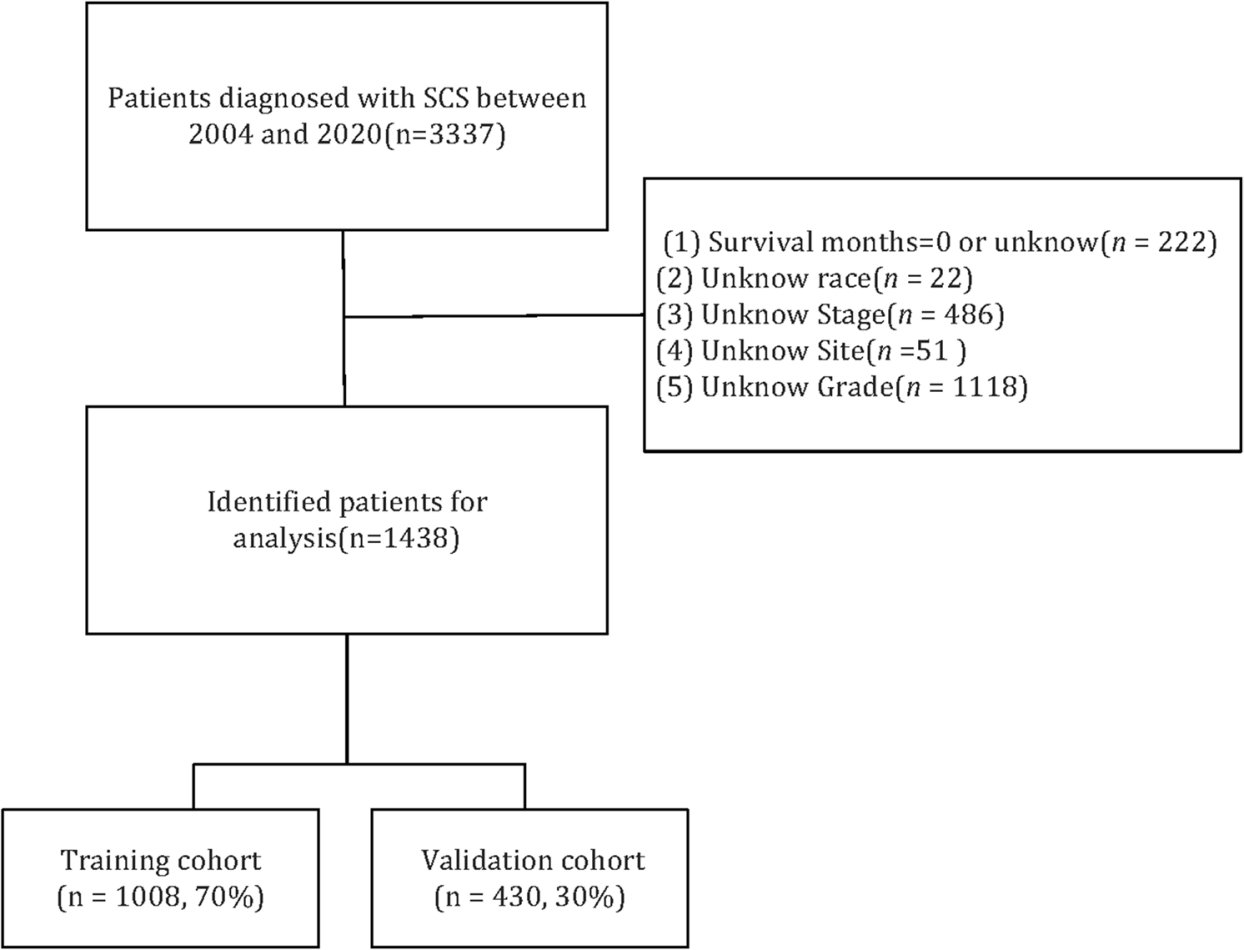
The flowchart of patient inclusion and division.

A total of 3337 patients with SCS between 2004 and 2020 were identified according to the International Classification of Disease for Oncology 3rd (ICD-O-3) code 8801/3, and SCS was confirmed to be the only primary malignancy. The exclusion criteria were as follows: (1) survival months = 0 or unknown, *n* = 222; (2) unknown race, *n* = 22; (2) unknown stage, *n* = 486; (4) unknown site, *n* = 51; and (5) unknown grade, n = 1118. Finally, 1438 patients were included in the total cohort. The flowchart of the selection process is shown in Fig. 1. The training cohort (n = 1008, 70%) and validation cohort (n = 430, 30%) were randomly assigned and generated. The SEER database is publicly available, so we did not need the approval of the institutional review board.

Age, sex, race, grade, SEER combined summary stage, primary tumour site, surgery, chemotherapy, radiotherapy, diagnosis year, survival months, vital status, and causes of death were obtained from the SEER database. The races included white, black, and others (American Indian/AK Native, Asian/Pacific Islander). Age was a continuous variable; it was transformed into a categorical variable according to the best cut-off methods of the X-tile program^22^, and the optimum cutoff points were determined (age, ≤ 59 years; 60–73 years, ≥ 74 years). The primary tumour locations were described as superficial organs (including skin and subcutaneous tissues) and deep organs (other organs). The cancer stages recorded according to the combined summary stage included localized, regional, distant, and unknown/unstaged. Surgery, chemotherapy, and radiotherapy were classified as yes or no/unknown and without a specific classification because the primary tumour site was not uniform. Survival time was calculated as the time interval from diagnosis of SCS to death from any cause or SCS and was recorded in months.

### Construction of the nomograms

Univariate and multivariate Cox analysis models were used to examine the potential predictors of both OS and CSS. First, univariate Cox analysis was used to screen for variables with p < 0.05 in the multivariate Cox proportional hazards regression analyses for both OS and CSS. Hazard ratios (HRs) and 95% confidence intervals (95% CIs) are listed. We established nomograms using these identified prognostic factors via multivariate Cox analysis for predicting 3- and 5-year OS and CSS in patients with SCS. The exact predictive performance and discriminative ability of the nomogram were evaluated using Harrell’s concordance index (C-index), area under the curve (AUC), and calibration curves in both the training and validation cohorts^23–25^. The differences in survival between patients stratified according to risk and treatment strategy in subgroups based on stage, location and grade were compared using Kaplan‒Meier curves and the log-rank test. The clinical utility and benefit of the nomogram were estimated by DCA ^26^. The X-tile package in R 4.3.0 included the R packages “rms”, “foreign”, “survival”, “survivalROC” and “survminer”, and P < 0.05 was considered to indicate statistical significance.

## Results

### Patient characteristics

A total of 1438 patients with SCS diagnosed from 2004 to 2020 were randomized into training (1008, 70%) and validation (430, 30%) cohorts. The clinicopathologic characteristics of the patients in the training and validation cohorts are shown in Table 1.

**Table 1:** Baseline demographic and clinical characteristics of SCS patients.

	Training cohort (N = 1008) (%)	Validation cohort (N = 430) (%)	Overall (N = 1438) (%)
Age(years)			
≤ 58	422 (41.9)	162 (37.7)	584 (40.6)
59–73	305 (30.3)	127 (29.5)	432 (30.0)
≥ 74	281 (27.9)	141 (32.8)	422 (29.3)
Race			
White	829 (82.2)	350 (81.4)	1179 (82.0)
Black	100 (9.9)	37 (8.6)	137 (9.5)
Others	79 (7.8)	43 (10.0)	122 (8.5)
Sex			
Male	527 (52.3)	213 (49.5)	740 (51.5)
Female	481 (47.7)	217 (50.5)	698 (48.5)
Grade			
I	57 (5.7)	18 (4.2)	75 (5.2)
II	218 (21.6)	73 (17.0)	291 (20.2)
III	277 (27.5)	124 (28.8)	401 (27.9)
IV	456 (45.2)	215 (50.0)	671 (46.7)
Stage			
Localized	508 (50.4)	209 (48.6)	717 (49.9)
Reginal	258 (25.6)	115 (26.7)	373 (25.9)
Distant	192 (19.0)	85 (19.8)	277 (19.3)
Unknow	50 (5.0)	21 (4.9)	71 (4.9)
Tumor location			
Superficial	748 (74.2)	315 (73.3)	1063 (73.9)
Deep	260 (25.8)	115 (26.7)	375 (26.1)
Surgery			
No	250 (24.8)	124 (28.8)	374 (26.0)
Yes	758 (75.2)	306 (71.2)	1064 (74.0)
Radiation			
No/unknow	545 (54.1)	226 (52.6)	771 (53.6)
Yes	463 (45.9)	204 (47.4)	667 (46.4)
Chemotherapy			
No	719 (71.3)	310 (72.1)	1029 (71.6)
Yes	289 (28.7)	120 (27.9)	409 (28.4)
Diagnosis year			
2004–2011	492 (48.8)	190 (44.2)	682 (47.4)
2012–2020	516 (51.2)	240 (55.8)	756 (52.6)

### Univariate and multivariate analyses

The univariate Cox model showed that age, grade, stage, tumour location, surgery, radiation, chemotherapy and diagnosis year were significant prognostic indicators for both OS and CSS, while the multivariate Cox proportional hazard analysis showed that age, stage, grade, tumour location, surgery, radiation and diagnosis year were independent prognostic factors for CSS, age, stage, grade, tumour location and surgery. The results of univariate and multivariate analyses of OS and CSS are listed in Tables 2 and 3.

**Table 2:** Univariate and multivariate analysis of OS in the training cohort of SCS patients.

	Univariate			Multivariate		
	HR	95% CI	P value	HR	95% CI	P value
Age (years)						
≤ 58	Reference			Reference		
59–73	1.87	1.54–2.28	< 0.01	1.91	1.56–2.34	< 0.01
≥ 74	2.67	2.20–3.24	< 0.01	2.90	2.34–3.56	< 0.01
Race						
White	Reference					
Black	0,92	0.70–1.20	0.53			
Others	1.24	0.93–1.64	0.14			
Sex						
Male	Reference					
Female	0.92	0.78–1.07	0.28			
Stage						
Localized	Reference			Reference		
Reginal	1.69	1.39–2.05	< 0.01	1.42	1.17–1.74	< 0.01
Distant	4.92	4.04–6.00	< 0.01	3.02	2.38–3.84	< 0.01
Unknow	2.09	1.46–2.97	< 0.01	1.36	0.94–1.99	0.10
Tumor location						
Soft tissue	Reference			Reference		
Others	1.48	1.25–1.76	< 0.01	1.35	1.13–1.62	< 0.01
Grade						
Grade I	Reference			Reference		
Grade II	1.04	0.67–1.62	0.85	0.97	0.63–1.52	0.91
Grade III	2.37	1.56–3.59	< 0.01	1.96	1.29–2.99	< 0.01
Grade IV	2.21	1.47–3.33	< 0.01	1.82	1.21–2.76	< 0.01
Surgery						
No	Reference			Reference		
Yes	0.24	0.20–0.28	< 0.01	0.35	0.29–0.43	< 0.01
Radiation						
No/Unknow	Reference			Reference		
Yes	0,77	0.66–0.90	< 0.01	0.83	0.71–0.99	0.04
Chemotherapy						
No	Reference			Reference		
Yes	1.65	1.40–1.95	< 0.01	1.12	0.92–1.35	0.25
Diagnosis year						
2004–2011	Reference			Reference		
2012–2022	0.84	0.72–1.00	0.04	0.82	0.70–0.97	0.02

**Table 3:** Univariate and multivariate analysis of CSS in the training cohort of SCS patients.

	Univariate			Multivariate		
	HR	95% CI	P value	HR	95% CI	P value
Age (years)						
≤ 58	Reference			Reference		
59–73	1.67	1.33–2.10	< 0.01	1.68	1.34–2.12	< 0.01
≥ 74	1.68	1.32–2.13	< 0.01	1.88	1.45–2.42	< 0.01
Race						
White	Reference					
Black	1.20	0.89–1.62	0.22			
Others	1.34	0.95–1.88	0.09			
Sex						
Male	Reference					
Female	0.95	0.78–1.15	0.57			
Stage						
Localized	Reference			Reference		
Reginal	1.96	1.53–2.51	< 0.01	1.58	1.23–2.04	< 0.01
Distant	7.06	5.56–8.90	< 0.01	3.82	2.87–5.06	< 0.01
Unknow	2.30	1.47–3.61	< 0.01	1.50	0.93–2.40	0.10
Tumor location						
Soft tissue	Reference			Reference		
Others	1.65	1.34–2.03	< 0.01	1.56	1.26–1.93	< 0.01
Grade						
Grade I	Reference			Reference		
Grade II	0.90	0.50–1.63	0.73	0.83	0.45–1.50	0.53
Grade III	2.85	1.65–4.94	< 0.01	2.28	1.31–3.97	< 0.01
Grade IV	2.62	1.52–4.50	< 0.01	2.08	1.20–3.59	< 0.01
Surgery						
No	Reference			Reference		
Yes	0.20	0.17–0.25	< 0.01	0.34	0.27–0.43	< 0.01
Radiation						
No/unknow	Reference			Reference		
Yes	0.80	0.66–0.97	0.03	0.89	0.73–1.09	0.27
Chemotherapy						
No	Reference			Reference		
Yes	2.20	1.81–2.67	< 0.01	1.20	0.96–1.50	0.11
Diagnosis year						
2004–2011	Reference			Reference		
2012–2022	0.82	0.68–1.00	0.04	0.84	0.69–1.02	0.08

### Construction and validation of the nomogram

**Figure 2:**
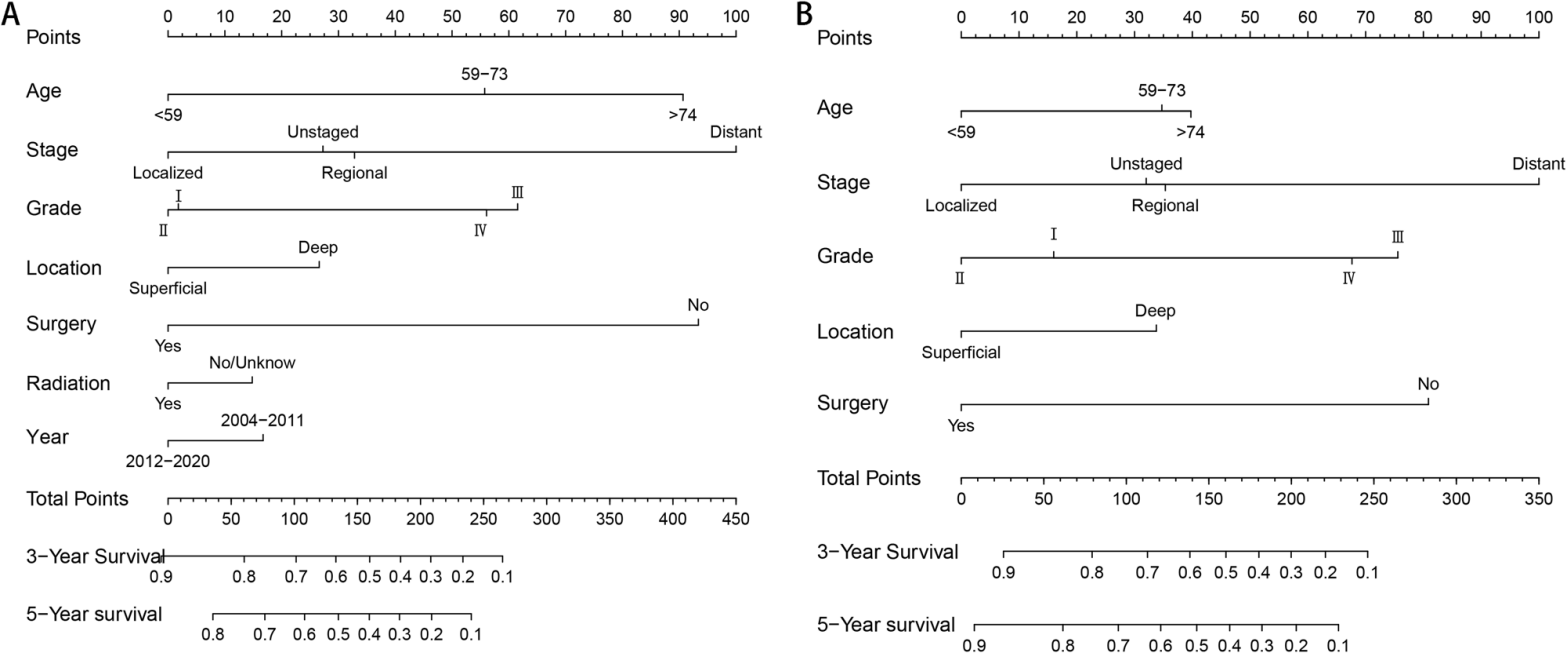
Nomograms for 3- and 5-year OS (**A**) and CSS (**B**) in patients with SCS.

**Figure 3:**
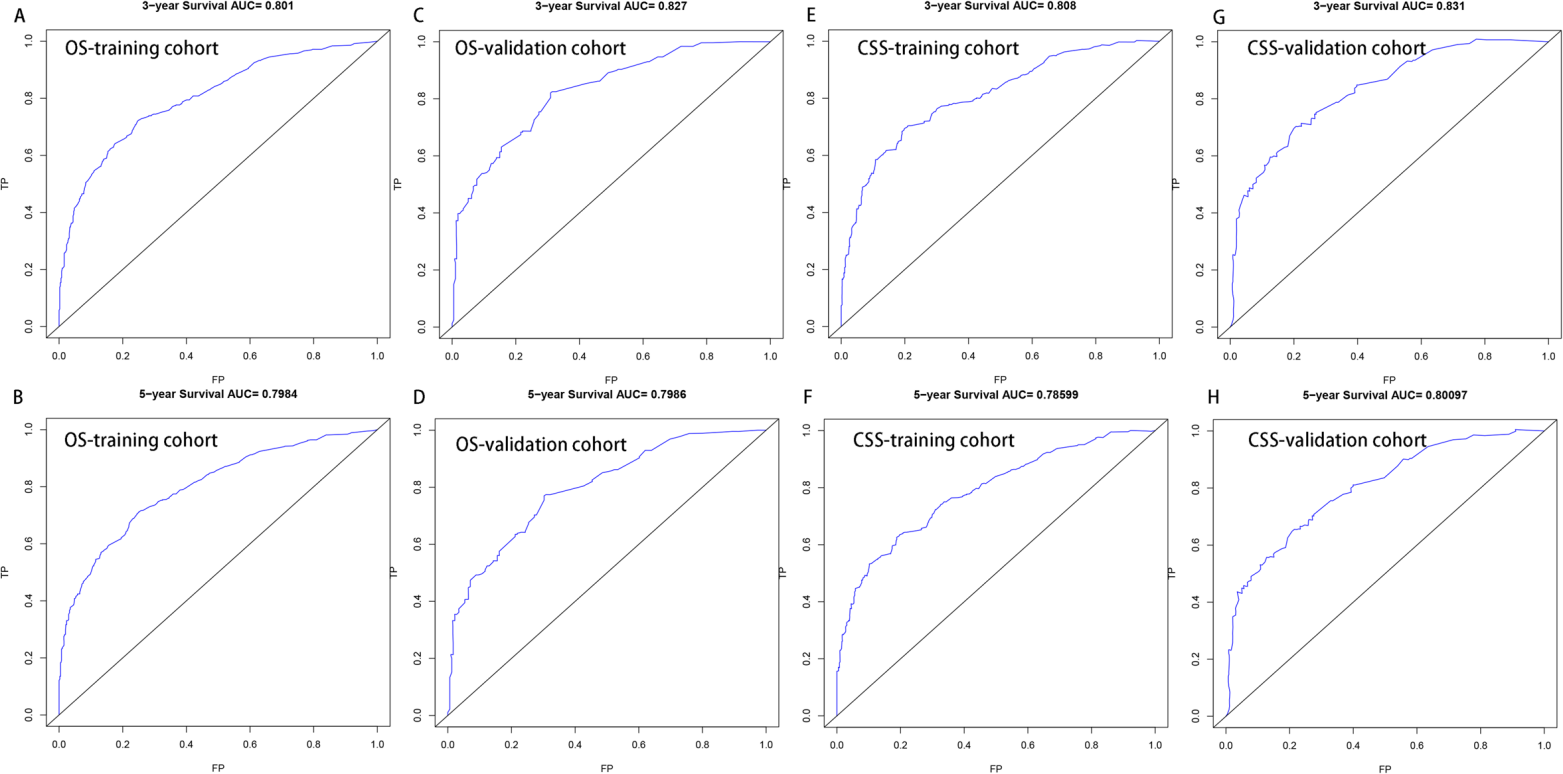
The ROCs for OS (**A–D**) and CSS (**E–H**) at 3 and 5 years in the training cohort and validation cohorts.

**Figure 4:**
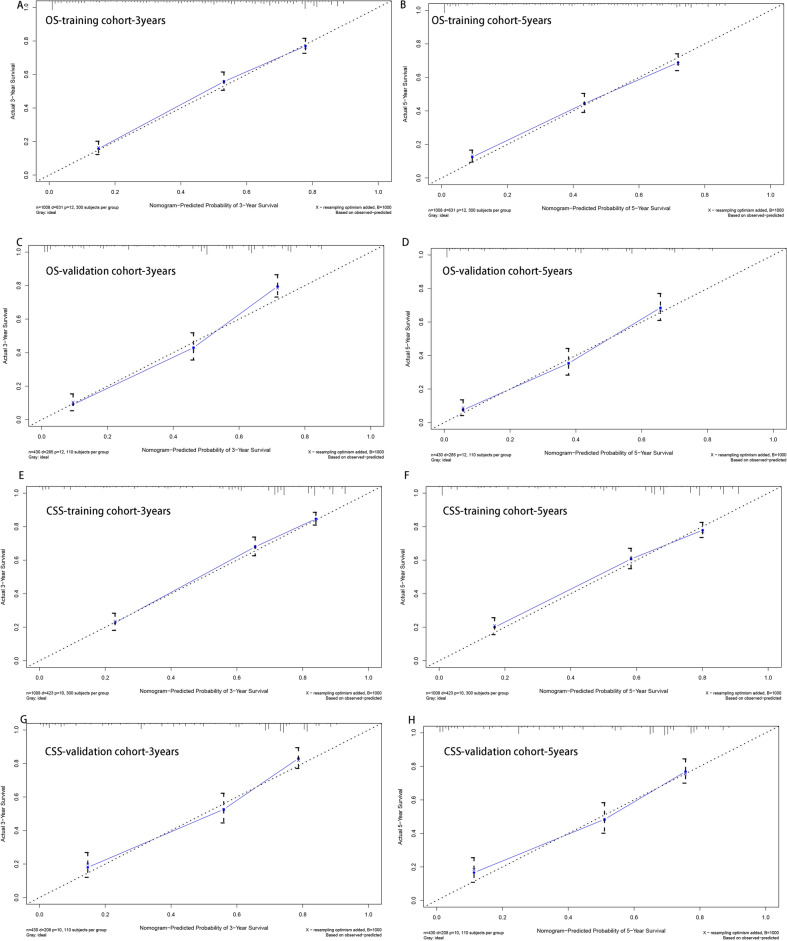
Calibration plots for 3- and 5-year OS in the training cohort (**A,B**) and validation cohort (**C,D**). Calibration plots for 3- and 5-year CSS in the training cohort (**E,F**) and validation cohort (**G,H**).

**Figure 5:**
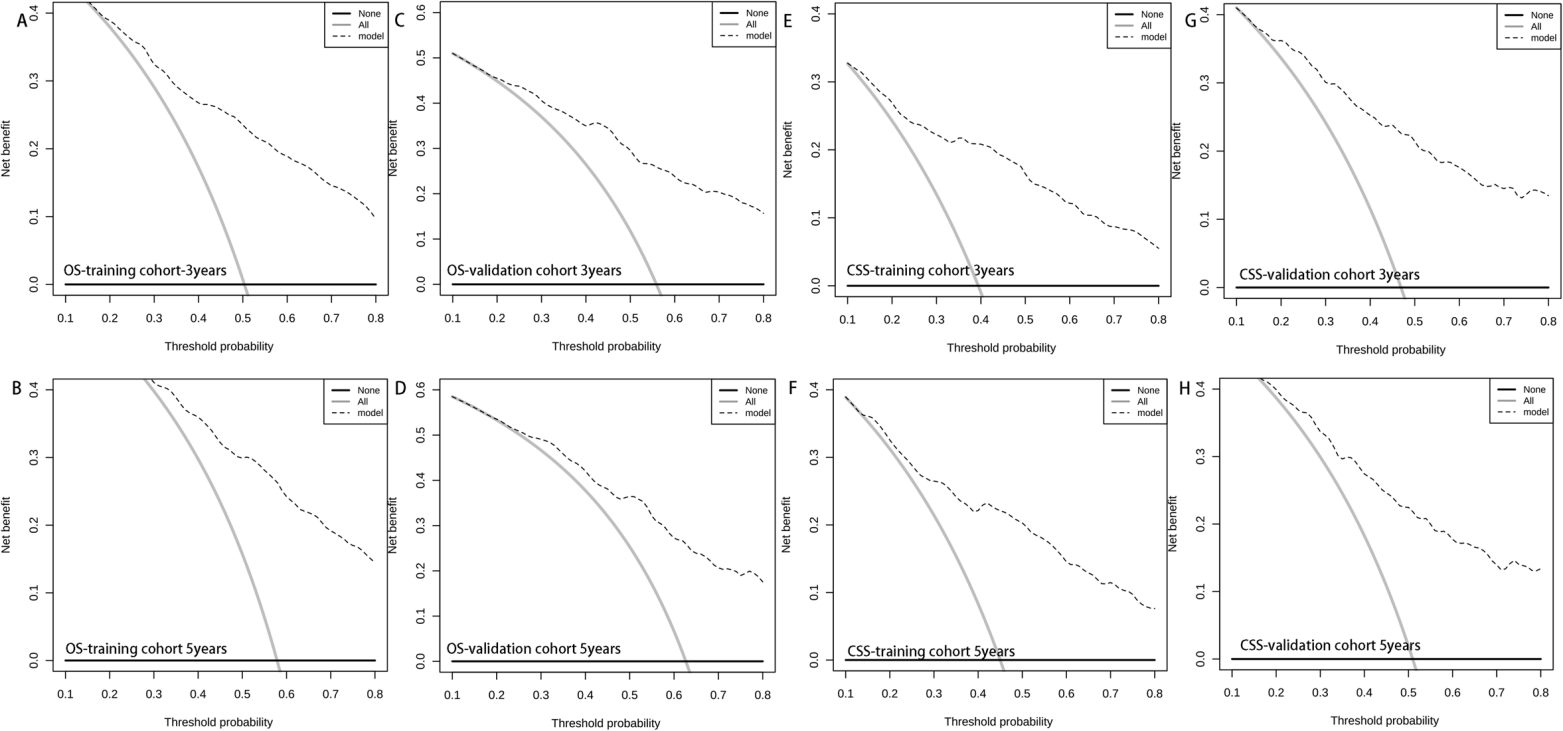
Decision curves of the nomogram for predicting OS in the training cohort (**A,B**) and the validation cohort (**C,D**). Decision curves of the nomogram for predicting CSS in the training cohort (**E,F**) and the validation cohort (**G,H**).

Then, nomogram models were established based on the results of multivariate Cox proportional hazard analysis for both OS (Fig. 2A) and CSS (Fig. 2B). The C-index of the OS predictive model was 0.76 in the training cohort and 0.77 in the validation cohort. For the CSS nomogram, the C-indices were 0.76 and 0.78 in the training cohort and validation cohort, respectively. Then, we evaluated the discriminatory ability of the nomogram by receiver operating characteristic (ROC) curve analysis. For OS, the 3/5-year AUCs were 0.801 and 0.798 in the training cohort (Fig. 3A, B) and 0.827 and 0.799 in the validation cohort (Fig. 3C, D). With respect to the CSS nomogram, the 3- and 5-year AUCs were 0.809 and 0.786, respectively, in the training cohort (Fig. 3E, F) and 0.831 and 0.901, respectively, in the validation cohort (Fig. 3G, H), which suggested the excellent discriminatory power of the models for both OS and CSS. Moreover, the 3- and 5-year calibration curves indicated that the nomogram was effective for both OS and CSS (Fig. 4). In addition, DCA was used to analyse the clinical practicality of the nomogram models in the training and validation cohorts for both OS and CSS; the results indicated that they had good positive and net benefits (Fig. 5).

### Risk classification system and Kaplan–Meier analysis of treatment efficacy

**Figure 6:**
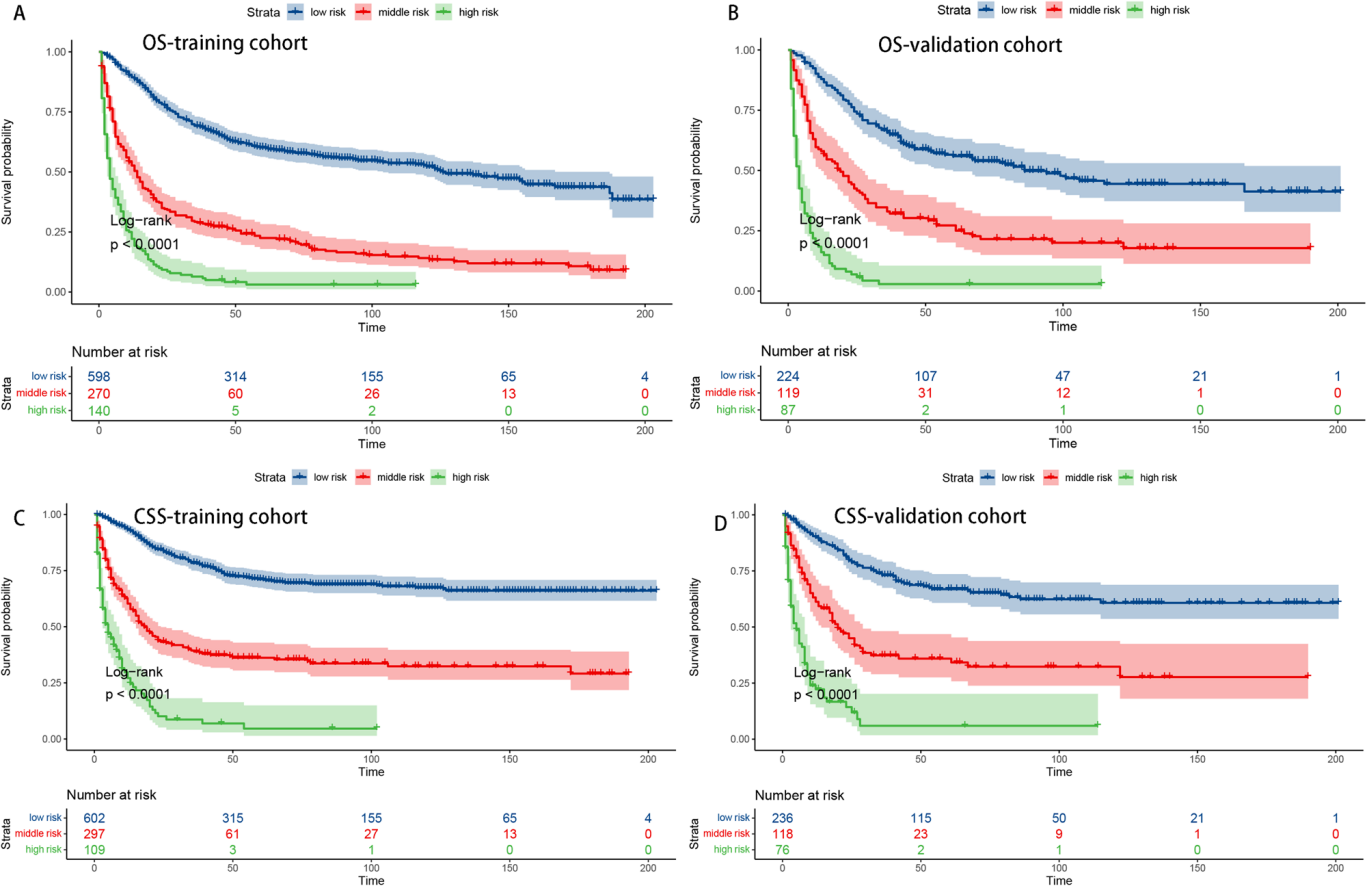
K‒M curves of OS in the low-, middle- and high-risk groups in the training set (**A**) and validation set (**B**). K‒M curves of CSS in the low-, middle- and high-risk groups in the training set (**C**) and validation set (**D**).

A risk classification system using the best cutoff determined by X-tile software was developed to further optimize the clinical application of the nomogram for both OS and CSS. Patients with SCS were categorized into three risk levels: the low-risk group, middle-risk group and high-risk group. We conducted Kaplan‒Meier curve and log-rank tests on these groups of patients based on risk stratification. The results demonstrated a consistent decline in survival outcomes, both in overall survival (OS) and cancer-specific survival (CSS), as the risk levels increased, confirming our initial expectations (Fig. 6A–D).

**Figure 7:**
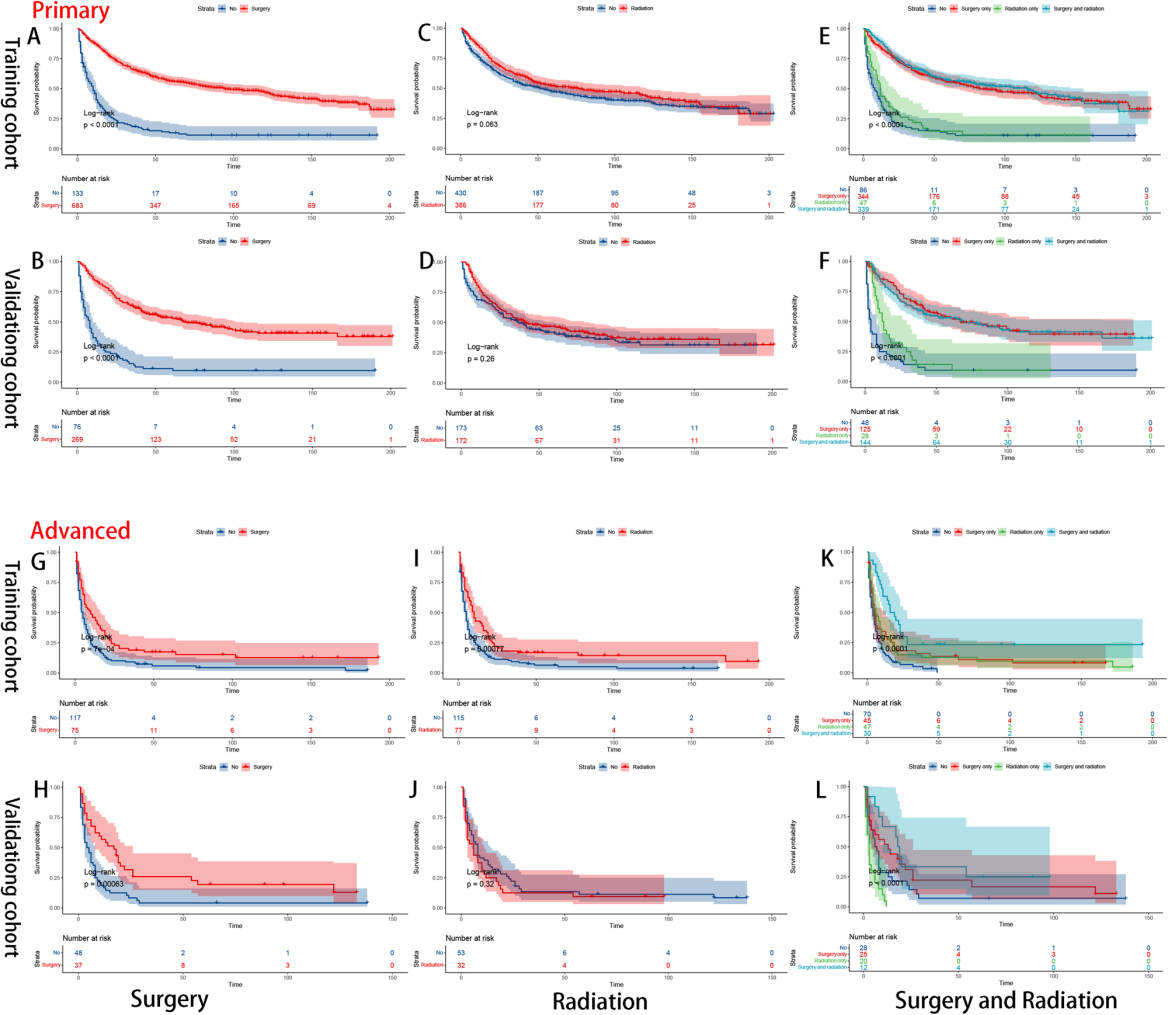
K‒M survival curves of OS stratified by stage subgroup [primary (**A–F**); advanced (**G–L**)].

**Figure 8:**
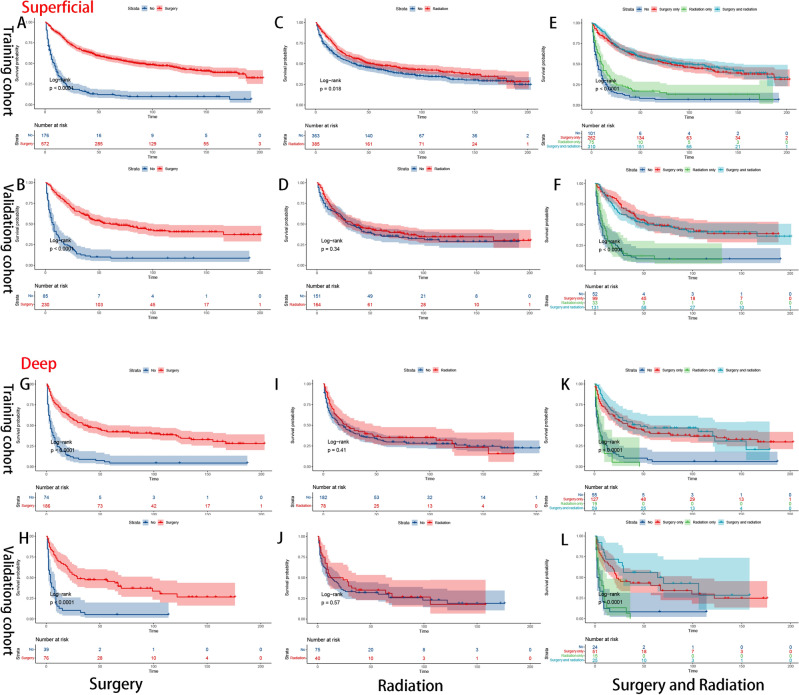
K‒M survival curves of OS stratified by location subgroup [superficial (**A–F**); deep (**G–L**)].

**Figure 9:**
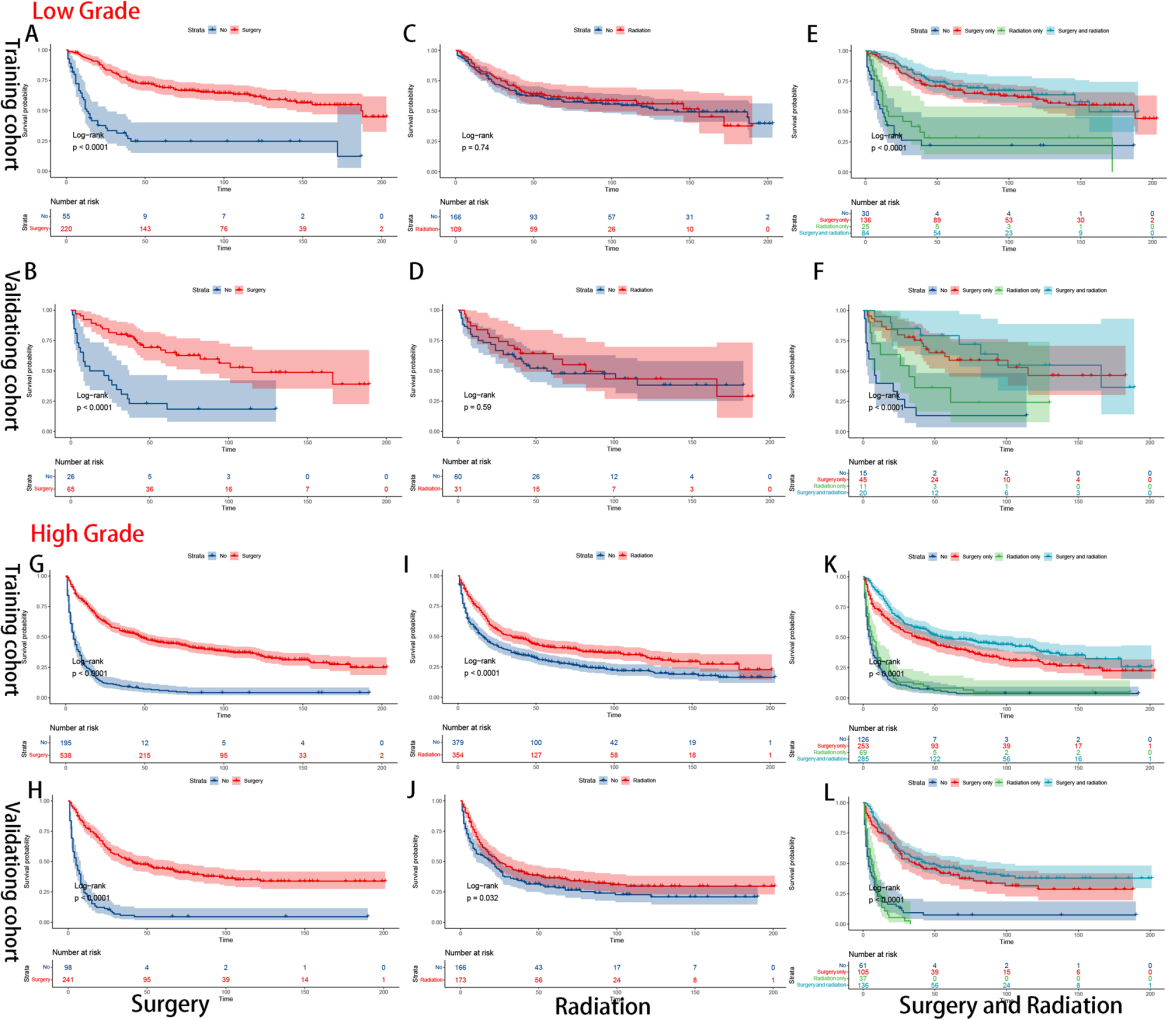
K‒M survival curves of OS stratified by tumour grade [low grade (**A–F**); high grade (**G–L**)].

The Kaplan–Meier method was used to assess treatment strategy grouping according to stage (Fig. 7), location (Fig. 8) and grade (Fig. 9). According to our aforementioned findings, surgery and radiotherapy emerged as independent prognostic factors for overall survival (OS). Therefore, we categorized the treatment modalities into four groups: no treatment, surgical treatment alone, radiation therapy alone, and combined surgery with radiation therapy. The results revealed that surgical intervention significantly impacted survival outcomes in all subgroups (Figs. 7A, B, G, H, 8A, B, G, H, 9A, B, G, H), whereas the impact of radiotherapy was comparatively limited. The clinical benefits of radiotherapy were exclusively observed in the high-grade groups (Fig. 9C, D, I, J). The combination therapy strategy demonstrated a prognostic advantage (P < 0.01)) in the high-grade subgroup when surgery was combined with adjuvant radiotherapy compared to surgery alone. However, there was no significant difference (P = 0.16) in clinical benefit between the radiotherapy alone group and the untreated group (Fig. 9E, F, K, L).

## Discussion

Recently, there have been increasing reports on the use of nomograms for predicting the prognosis of patients with various sarcomas^18,27,28^. The advantage of this approach is that by combining various independent risk factors based on the patient's condition, the prognosis can be more intuitively evaluated and personalized, and the OS and CSS can be quantified individually, allowing more accurate prognosis prediction^19^. For the first time and by means of this study, nomograms have been established for predicting the prognosis of SCS patients.

SCS is an extremely rare sarcoma for which there is almost no clinical evidence indicating prognosis. Therefore, we constructed a nomogram to predict the prognosis of SCS patients. A high area under the ROC curve indicated that the nomogram accurately predicted the probability of 3-OS, 5-OS, or CSS in SCS patients (0.79–0.83). Calibrating the curve indicated a high degree of consistency between the predicted and actual survival rates. Survival analysis according to demographic characteristics indicated that sex and race were not independent prognostic indicators for CSS or OS in patients with SCS, which is consistent with previously published results^21^. By measuring the standard deviation at the nomogram scale, we found that for both OS and CSS, age, stage, grade, location and surgery were the most important prognostic factors.

The prognosis tends to worsen with age, which is consistent with what has been observed in the majority of other cancers. The SEER database defines tumour staging as follows: localized (tumour confined to the organ without invasion of surrounding tissues or lymph node metastasis), regional (tumour invading the organ or with lymph node metastasis), or distant (distant metastasis). The prognosis of patients with SCS deteriorates as tumour stage progresses, particularly in the distant stage. Subsequent treatment strategy studies will assign the first two groups to the primary group, while the distant group will be allocated to the advanced group.

The histopathological grade of tumours can reflect the degree of abnormality between tumour cells and normal tissues and is an indicator of tumour growth and spread. It is usually closely related to the prognosis in tumour patients^29,30^. Based on our research findings, patients diagnosed with Grade IIII (poorly differentiated) or Grade IV (undifferentiated) gliomas exhibit a significantly inferior prognosis in comparison to those diagnosed with Grade I (well differentiated) or Grade II (moderately differentiated) gliomas. According to the SEER data we extracted, SCS is commonly found primarily in the skin and subcutaneous soft tissues of the limbs and trunk and rarely in organs and deep tissues such as the peritoneum, parotid gland, spleen, liver, heart, and lung. We divided the primary locations of the tumour into superficial and deep tissue. We found that regarding SCS, patients in the superficial tissue group had a better prognosis than those in the deep organ group in terms of both OS and CSS, consistent with the findings of previous research^21^.

Most of the reported cases of SCS involved multimodal treatment, including surgical management, radiation therapy, and chemotherapy^7,31–33^. Surgery is the main treatment method combined with adjuvant therapy, and primary chemotherapy or radiation therapy is generally used only for patients with unresectable or widely metastatic tumours^34,35^. In this study, we concluded that SCS patients in the surgical group achieved good prognostic outcomes in terms of OS and CSS in patients, consistent with the findings of previous studies^21^. This association was found to be particularly pronounced within the primary group as we further stratified the data into subgroups based on stage. Moreover, upon categorizing surgery and radiation therapy into subgroups, we found that combining surgery with adjuvant radiation therapy yielded superior prognostic benefits compared to surgery alone for patients with high tumour grades. The pathological grade of the tumour and the presence of negative or positive margins following surgical resection have been extensively investigated, as they play pivotal roles in determining whether surgical patients should receive adjuvant radiation therapy or chemotherapy ^36–38^. As the primary site of SCS is distributed throughout the body, it is not feasible to discuss specific surgical, radiotherapy or chemotherapy methods^21^. The present study was stratified based on the depth of the tumour location, and both cohorts demonstrated that surgery was of considerable clinical importance, whereas radiotherapy failed to confer any prognostic benefits.

The X-tile algorithm allows us to perform a very reliable analysis of the optimal cutoff point and determine the optimal cutoff value for age ^22^ as we did in this study. This program can also be used to create a risk stratification system based on survival rate and has been used for survival analysis of many malignant tumours, such as gastric cancer^39^, colon cancer^40^, renal carcinoma^41^, and pancreatic ductal adenocarcinoma^42^. In this study, a risk stratification system with three risk groups consistently showed significant differences in the KM survival curves for both OS and CSS in the training group and validation group, demonstrating the effectiveness of the risk stratification system.

The limitations of this article are summarized as follows: (1) external validation was not conducted and was difficult to achieve due to the extremely low incidence rate of SCS; additional large multicentre studies may need to be performed; (2) the surgical method and chemotherapy regimen were not specified but were classified as "yes" or "no"; This issue must be discussed based on the specific site of the primary tumour, and further research can be conducted; (3) the SEER database does not record smoking history, drinking history or other personal history; moreover, hypertension, diabetes and other basic diseases may influence the prognosis of SCS patients^43^.

## Conclusion

In summary, based on the large number of SCS samples in the SEER database, we established and validated new nomograms to predict the prognosis of SCS patients in terms of both OS and CSS via the R package 4.3.0. This research will help doctors more precisely evaluate the prognosis of SCS patients and help in the formulation of treatment strategies.

## Data Availability

The datasets generated and analysed during the current study are available in the SEER database [https://seer.cancer.gov/].

## Abbreviations

SCS: Spindle cell sarcoma
SEER: Surveillance, Epidemiology, and End Results
OS: Overall survival
CSS: Cancer-specific survival
C-index: Concordance index
ROC: Receiver operating characteristic
AUC: Area under the receiver operating characteristic curve
DCA: Decision curve analysis.
ICD-O-3: International Classification of Disease for Oncology, Third Edition
